# Town and Village Typhoid

**Published:** 1902-08-30

**Authors:** 


### TOWN AND VILLAGE TYPHOID.

An outbreak of typhoid fever, which was evidently unconnected with water supply, led Dr. Veeder[1] to inquire into the sources other than water from which the infection of this disease might be derived. In regard to infection from the soil, he came to the conclusion that this might be conveyed to the stomach in various ways; vegetables, which are eaten raw, such as radishes, lettuce and celery, if insufficiently cleansed, will surely convey infection from the soil; so, too, will dust when containing desiccated typhoid excreta, or even merely desiccated typhoid bacilli descended from excreta, if there should be any wind; flies, again, as is well known, may carry the infection. Moreover, it must not be supposed that mere burying of typhoid excreta will prevent the spread of infection, for the natural tendency of the bacilli is to grow up towards the surface, and if it be true, as is stated, that in foul soil they can retain their vitality for a year or more, it is quite possible for buried excreta to become a source of infection. While admitting that in cities typhoid is often water-borne, he holds that in villages and country places it is far more often carried by dust, flies, and unclean food, while even in cities, in which the diminution of typhoid has followed the provision of a public water supply, he thinks that very often the improvement has been due not so much to the increased purity of the water as to the coincident introduction of water-closets. "It is improper construction of closets that starts many of the epidemics of typhoid in villages and the suburbs of cities that are commonly ascribed to bad water. It is true that on the introduction of a public system of water supply epidemics of this particular type cease, but this is due to substitution of plumbing for such miserable closet arrangements rather than to any change in the water." In reference to the proposition laid down by Dr. Veeder, namely, that "in villages and camps typhoid is usually fly-borne and in cities water-borne," it would evidently be a matter of considerable concern to ascertain whether there is any difference between the seasonal prevalence of village and of town typhoid; for village typhoid ought to correspond more especially with the prevalence of such warm weather as should encourage the multiplication and wandering of house flies, while town typhoid ought more particularly to prevail when storms following prolonged drought carry accumulations of infected matter into the water courses from which public water supplies are derived. That town typhoid often follows downpours of rain has been shown in many cases. What we now want to know is whether village typhoid more particularly coincides with dryness and warmth.

[1] New York Medical Record, July 26th.

